# Risk of metastasis in *BRCA2* carriers diagnosed with triple‐negative breast cancer

**DOI:** 10.1002/cam4.6267

**Published:** 2023-07-23

**Authors:** Marcelo Moreno, Júlia Salles Oliveira, Rafael Canfield Brianese, Douglas Guedes de Castro, Solange Moraes Sanches, Giovana Tardin Torrezan, Karina Miranda Santiago, Marina De Brot, Vladmir Claudio Cordeiro de Lima, Fabiana Baroni Alves Makdissi, Jose Claudio Casali Da Rocha, Vinicius Fernando Calsavara, Dirce Maria Carraro

**Affiliations:** ^1^ Graduate Program of A.C. Camargo Cancer Center São Paulo Brazil; ^2^ Medicine Course and Biomedical Sciences Federal University of Fronteira Sul Chapecó Santa Catarina Brazil; ^3^ Clinical and Functional Genomics Group CIPE, A.C. Camargo Cancer Center São Paulo Brazil; ^4^ Department of Radiation Oncology A.C. Camargo Cancer Center São Paulo Brazil; ^5^ Department of Medical Oncology A.C. Camargo Cancer Center São Paulo Brazil; ^6^ National Institute of Science and Technology in Oncogenomics and Therapeutic Innovation (INCITO) São Paulo Brazil; ^7^ Department of Anatomic Pathology A.C. Camargo Cancer Center São Paulo Brazil; ^8^ Department of Breast Surgery A.C. Camargo Cancer Center São Paulo Brazil; ^9^ Department of Oncogenetics A.C. Camargo Cancer Center São Paulo Brazil; ^10^ Biostatistics and Bioinformatics Research Center Cedars‐Sinai Medical Center Los Angeles California USA

**Keywords:** BRCA mutations, *BRCA1* gene, *BRCA2* gene, breast cancer, metastasis

## Abstract

**Background:**

Triple‐negative breast cancer (TNBC) is the neoplasia most associated with *BRCA1* germline pathogenic variants (PV) and is more likely to develop metastases than the other breast cancer (BC) subtypes, mainly in the lungs and the central nervous system (CNS). Recently, *BRCA2* carriers were shown to have a higher risk for developing CNS metastases. However, the patterns of recurrence and metastases of *BRCA2* carriers with TNBC are unknown.

**Methods:**

TNBC patient data attending the A.C. Camargo Cancer Center, from 1998 through 2020, were verified either by medical records or by *BRCA1/2* genetic testing carried out. Multivariable logistic regression models were fit to the data to assess the independent factors for bone and CNS metastases. Adjustment was done using all independent variables with *p* < 0.2 in the univariable Cox model to describe the relationship between the independent variables until time of death.

**Results:**

A total of 388 TNBC patients were evaluated. We identified PV in *BRCA1/2* genes in 21% (82/388), being 17.7% (69/388) in *BRCA1* and only 3.3% (13/388) in *BRCA2*. A total of 120 patients (31%) developed distant metastases. Bone or CNS metastases were observed in 40% and 60% of *BRCA2* PV carriers (*p* = 0.155), respectively. The *BRCA2* carriers tended to have a higher likelihood of developing bone metastases (OR, 4.06; 95% CI, 0.82–20.01; *p* = 0.085), when compared to *BRCA1* carriers (OR, 0.6; 95% CI, 0.12–2.87; *p* = 0.528). *BRCA2* carriers had an OR of 1.75 (95% CI, 0.33–9.14; *p* = 0.503) for CNS metastasis development, while *BRCA1* carriers had an OR of 0.72 (95% CI, 0.23–2.23; *p* = 0.574).

**Conclusions:**

Patients with TNBC and PV in the *BRCA2* gene had higher frequencies of secondary bone involvement and CNS in the course of the disease. However, the *BRCA2* PV did not represent an independent outcome predictor of metastases and overall survival. Efforts to increase the number of *BRCA2* carriers among TNBC patients are crucial for determining their risk of developing bone and CNS metastases compared to *BRCA2* noncarriers.

## Graphical abstract

The association of germline pathogenic variants in BRCA2 gene and triple‐negative breast cancer (BC) is uncommon. We evaluated 388 patients triple‐negative BC from a single institution to verify the risk of metastasis in BRCA2 carriers. Patients with triple‐negative BC and *BRCA2* pathogenic variants had higher frequencies of metastases bone and central nervous system.

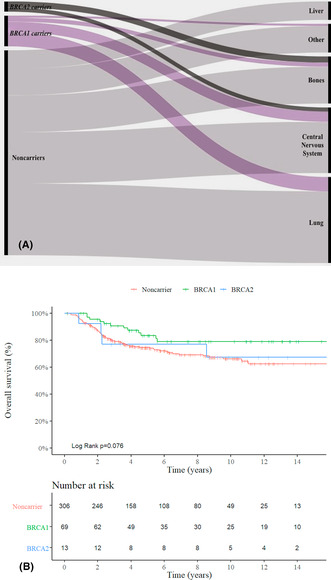

## 1 INTRODUCTION

The triple‐negative breast cancer subtype (TNBC) accounts for about 15% of breast cancers (BC) and frequently affects young women (<40 years old), with a predominance among African‐descent women in the United States.^1^, ^2^, ^3^ TNBC has a poor prognosis, due to early recurrence, high proclivity to develop metastases mainly to the lungs and central nervous system (CNS), and a lack of available targeted therapy.^1^, ^4^ The median survival of patients with stage IV TNBC ranges from 6.8 to 13.3 months.^5^ This more aggressive biological behavior, often without a well‐established pattern, is due to the diversity of TNBC subtypes, which results in different response rates to treatments.^1^, ^6^

About 5%–10% of all BC cases are hereditary, mostly attributed to germline pathogenic variants (PV) in *BRCA1* or *BRCA2* genes (*BRCA1/2*). Patients who develop TNBC may carry a germline PV in the *BRCA1* gene, observed in up to 30% of individuals (*BRCA1* carriers) depending on the population studied. On the contrary, among *BRCA1* carriers that develop BC, the TNBC subtype is found in around 80% of patients. The association of germline PV in *BRCA2* (*BRCA2* carriers) and TNBC is uncommon (ranging from 2% to 16%), being more frequently associated with ER‐positive BC.^7^, ^8^, ^9^, ^10^, ^11^, ^12^, ^13^, ^14^, ^15^, ^16^

In a previous study, where patients with all immunohistochemical subtypes of BC were investigated for the existence of a germline PV in *BRCA1/2*, it was observed that *BRCA1/2* carriers were more prone to develop CNS metastasis compared to patients that did not carry PVs in these genes (noncarriers).^17^ When controlled by BC subtypes, this association was higher among *BRCA2* carriers with TNBC. However, the metastasis patterns in patients with TNBC and *BRCA2* carriers are not clear.^17^, ^18^ Therefore, we propose to verify the metastatic pattern and survival rate in patients with TNBC that bear PV in *BRCA2* gene, in comparison with that observed in *BRCA1* carriers and noncarriers.

## 2 MATERIALS AND METHODS

### 2.1 Patient data

After obtaining institutional review board approval, patients with TNBC diagnosed and treated at A.C. Camargo Cancer Center (ACCCC), in São Paulo, Brazil, were retrospectively identified and clinicopathological data were obtained from electronic medical registers between November 1998 and March 2020. TNBC status was considered as stated in the records, which was negative for ER and PR if less than 1% of neoplasia cell nuclei were immunoreactive; for HER2, negative cases included those scored on immunohistochemistry as 0 and 1+. For score 2+/equivocal carcinomas, cases with an in situ hybridization test result confirming the absence of *HER2* gene amplification were also considered. Patients were selected based on genetic testing for *BRCA1*/*BRCA2* genes availability in the digital medical records or based on available DNA samples from leukocytes, adjacent normal tissue or tumor tissue provided by the ACCCC Biobank. We performed genetic testing in DNA obtained from leukocyte/fresh frozen normal tissue using a panel of 27 cancer‐predisposing genes (customized IDT—Integrated DNA Technologies). For patients with only tumor tissue available, somatic analysis was realized in formalin‐fixed paraffin‐embedded neoplasia samples using a 15‐gene panel (Oncomine BRCA Expanded Panel—Thermo Fisher). Germline tests were sequenced on the MiniSeq (Illumina) or NextSeq 500 (Illumina) platforms, and, somatic tests were performed on the Ion S5 System (Thermo Fisher). For germline testing, the mapping and calling of variants were performed and data were analyzed using the VarSeq software (v2.1.1/GoldenHelix) according to the filters: coverage ≥50×, allelic frequency ≥20%, SNVs and indels present in the coding region or close to the splicing site (−20 and +20). For somatic testing, data analyses were performed using the Torrent Suite (v.5.12.3/Termo Fisher) and Ion Reporter (v8.18.2.1/Thermo Fisher), (workflow Oncomine BRCA Expanded—540—w4.2—DNA—Single Sample) software with the following criteria: >90% of target regions coverage 500×, variants within the coding sequence, and, that affect protein sequences (missense, nonsense, splice site alterations, and indels), not reported or showing a minor allele frequencies <1% in populational databases (dbSNP, gnomAD, ExAC). Germline PVs were considered when allelic frequency was higher than 40%. For variants reported in the ClinVar public database, the classification provided was considered. For variants that were not reported in ClinVar, or that were reported as conflicting or as variant of uncertain clinical significance (VUS), the information was checked in VarSome, (a public automated classification tool, according to the recommendations of the American College of Medical Genetics and Genomics—ACMG), and the classification of the latter was considered.^19^ Male patients and patients whose genetic test results were found inconclusive were excluded. The clinical variables evaluated were as follows: date of initial diagnosis, initial staging, age at diagnosis (date of the first biopsy with TNBC result), self‐declared ethnicity (black, brown, white or Asian), *BRCA* status (when available), tumor characteristics (histology and tumor grade), treatment history, date of first metastatic event, anatomical site affected with metastasis and date of death or last follow‐up. The treatment was categorized “yes” or “no” for neoadjuvant chemotherapy, adjuvant chemotherapy, adjuvant radiotherapy, and surgery. Metastatic cases were considered when indicated as such by imaging tests or physiological changes reported by the treating physician in the patient's medical record. All distant site metastases were considered as distant metastases, except for the infraclavicular and supraclavicular lymph nodes, contralateral breast, locoregional skin, and chest muscle wall. CNS recurrence was defined as a distant metastatic lesion that occurred in the encephalic mass and meninges.

### 2.2 Statistical methods

Descriptive analysis was performed using the counts and percentages for categorial variables and mean, standard deviation (SD), medians, and ranges for continuous variables. Bivariate analysis was done to evaluate possible association between the independent variables with bone/CNS metastasis using the chi‐square test or Fisher's exact test for the categorical variables. Univariable and multivariable analyses for the occurrence of metastasis were performed using a logistic regression model separately for each anatomical site. Adjustment was done using clinical characteristics as T, N, initial stage and neoadjuvant chemotherapy. We evaluated the goodness of fit using the Hosmer–Lemeshow test. In addition, the analyses of the time to death were performed using the Kaplan–Meier curves and the survival curves were compared applying the log‐rank test. Univariable and multivariable Cox regression models were performed, and the proportional hazards assumption was assessed with Schoenfeld's residuals. Adjustment was done using all independent variables with *p* < 0.2 in the univariable Cox model. No imputation method was used for missing data. All analyses were conducted using R software version 4.0 with two‐sided tests at a significance level of 0.05.

## 3 RESULTS

### 3.1 Population characteristics

Data from 400 patients with TNBC were retrieved and perused for mutations in *BRCA1/ BRCA2* genes, and were classified as “*BRCA1* carriers,” “*BRCA2* carriers,” and “*BRCA1/2* noncarriers.” Twelve patients were excluded due to sequencing failure (12 patients); thus, 388 remaining patients were analyzed. Of those, 17.7% (69/388) were *BRCA1* carriers, 3.3% (13/388) were *BRCA2* carriers, and 76.5% (297/388) were *BRCA1/2* noncarriers. The nine patients (2.3%) that had VUS and were grouped with the noncarriers for further analysis. The mean age at diagnosis was lower in *BRCA1* carriers (41.6 y) than in *BRCA2* (46 y) carriers and in *BRCA1/2* noncarriers (48 y) (*p* < 0.001). There were no differences on the self‐declared ethnicity were observed, as most of the *BRCA1* carrier, *BRCA2* carrier and *BRCA1/2* noncarriers cases were self‐declared as white (*p* = 0.260). Both *BRCA1/2* carrier and noncarrier patients had tumors classified as G2 or G3, as expected for TNBC (*p* = 0.793). Most patients had invasive ductal carcinomas, regardless of whether they were *BRCA1/2* carriers or not. There were no cases of invasive lobular carcinoma diagnosed in patients with PV (*p* = 0.249). The tumor stage (T) classification was predominantly T2 for *BRCA1* carriers and noncarriers, while for *BRCA2* carriers, T2 and T3 were more common (*p* = 0.260). Considering locoregional lymph node involvement, most noncarrier patients and *BRCA1* carriers had no lymph node involvement at diagnosis, while *BRCA2* carriers had proportionally more N1 and N3 disease (*p* = 0.049). Regarding the use of neoadjuvant chemotherapy, surgery for primary BC, and adjuvant treatment, there was no difference between PV carrier and noncarrier patients. Considering the systemic treatment, 4.3% (13/305) noncarriers, 4.3% *BRCA1* carriers, and 0.0% (0/12) *BRCA2* carriers with available information did not receive neoadjuvant and adjuvant chemotherapy. Some patients received adjuvant chemotherapy only, other patients received neoadjuvant chemotherapy only, and some received both, which is why percentages of patients receiving adjuvant and neoadjuvant chemotherapy do not sum up. All *BRCA2* carriers that did not receive adjuvant chemotherapy were treated with neoadjuvant chemotherapy except for one for whom we do not have information (Table 1).

**TABLE 1:** Patient and triple‐negative neoplasm data according to BRCA Germline status (*N* = 388).

Variables	No. of patients (% BRCA status)			*p*‐value
	*BRCA1* carriers	*BRCA2* carriers	Noncarriers	
	*N* = 69, *n* (%)	*N* = 13, *n* (%)	*N* = 306, *n* (%)	
Age (years)				
≤40	36 (52.2)	2 (15.4)	99 (32.4)	0.002**
>40	33 (47.8)	11 (84.6)	207 (67.6)	
Ethnicity				
Asian	1 (1.9)	1 (11.1)	8 (2.6)	0.260
Black	0 (0.0)	1 (11.1)	10 (3.2)	
Brown	5 (9.6)	1 (11.1)	25 (8.1)	
White	46 (88.5)	6 (66.7)	182 (59.4)	
Unknown	17 (23.9)	4 (30.8)	81 (26)	
Tumor grade				
G1	1 (1.7)	0 (0.0)	3 (1.0)	0.793
G2	16 (26.7)	4 (33.3)	76 (25.9)	
G3	43 (71.7)	8 (66.7)	214 (73.0)	
Unavailable	9 (12.7)	1 (7.7)	13 (4.4)	
Histology				
Ductal	59 (89.4)	9 (74.9)	258 (85.0)	0.249
Lobular	0 (0)	0 (0)	8 (2.6)	
Mixed	2 (3.0)	2 (16.7)	24 (7.9)	
Other^a^	5 (7.6)	1 (8.3)	14 (4.6)	
Unavailable	3 (4.2)	1 (7.7)	2 (0.7)	
Tumor classification				
T1	11 (16.7)	1 (8.3)	46 (15.0)	0.220
T2	38 (57.6)	4 (33.3)	160 (52.2)	
T3	8 (12.1)	4 (33.3)	38 (12.4)	
T4	6 (9.1)	2 (16.7)	45 (14.7)	
TX	3 (4.5)	1 (8.3)	6 (2.0)	
Unavailable	3 (4.2)	1 (7.7)	11 (3.5)	
Lymph node classification				
N0	34 (51.5)	4 (33.3)	139 (45.4)	0.049*
N1	16 (24.2)	5 (41.7)	88 (29.7)	
N2	10 (15.2)	0 (0.0)	35 (11.4)	
N3	4 (6.1)	1 (8.3)	19 (6.2)	
NX	2 (2.8)	2 (15.3)	1 (4.1)	
Unavailable	3 (4.2)	1 (7.7)	13 (4.2)	
AJCC Stage				
I	12 (19.0)	1 (8.3)	32 (11.1)	0.349
II	33 (52.4)	5 (41.7)	154 (53.7)	
III	16 (25.4)	4 (33.3)	81 (28.2)	
IV	2 (3.2)	2 (16.7)	20 (7.0)	
Unavailable	6 (8.5)	1 (7.7)	19 (6.4)	
Neoadjuvant chemotherapy				
Yes	33 (50.8)	7 (58.3)	161 (52.6)	0.816
No	32 (49.2)	5 (41.7)	132 (43.1)	
Unavailable	4 (5.7)	1 (7.6)	14 (4.5)	
Surgical treatment of primary tumor				
Yes	66 (98.5)	12 (92.3)	297 (97.1)	1.00
No	1 (1.5)	1 (7.6)	9 (2.9)	
Unavailable	2 (2.8)	0 (0)	0 (0)	
Adjuvant chemotherapy				
Yes	44 (67.7)	4 (30.7)	184 (60.1)	0.723
No	21 (32.3)	4 (30.7)	110 (36.0)	
Unavailable	4 (5.7)	5 (38.4)	12 (4.0)	
Adjuvant radiotherapy				
Yes	50 (72.4)	9 (69.2)	225 (73.5)	0.986
No	15 (21.7)	3 (2.3)	67 (21.8)	
Unavailable	4 (5.7)	1 (7.6)	14 (4.5)	

^a^ Metaplastic carcinoma, adenoid cystic carcinoma, spindle carcinoma, papillary carcinoma.

* *p* < 0.05

** *p* < 0.01.

### 3.2 Pattern of distant metastasis

**FIGURE 1:**
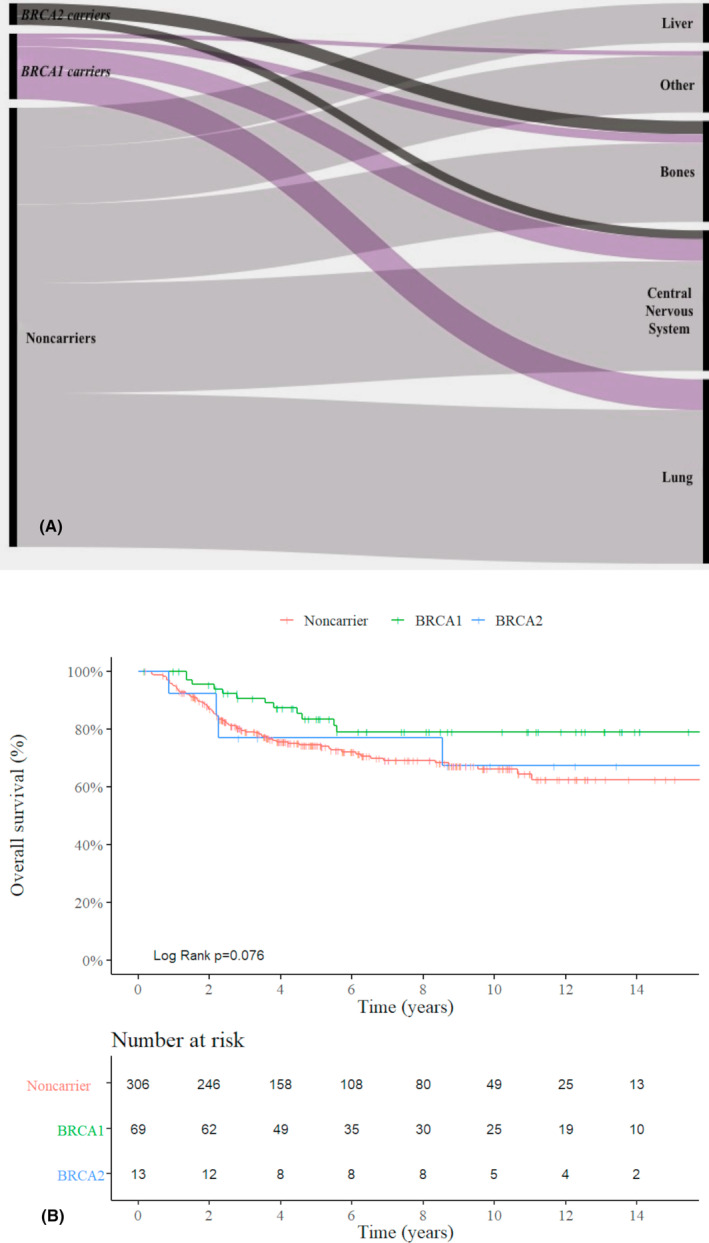
Triple‐negative breast cancer patients according to *BRCA* status. (A) Proportional First Visceral Metastasis (*N* = 120). (B) Kaplan–Meier curve of overall survival (*N* = 388).

Of the 388 patients, 120 (31%) patients developed metastases in distant organs (21.7%, 38.5%, and 32.7% in *BRCA1* carries, *BRCA2* carriers, and noncarriers, respectively) (Figure 1A and Table 2). *BRCA2* carriers had visceral metastases later when compared to the *BRCA1* carrier and noncarrier groups (*p* = 0.027) (Table 2). Most noncarriers and *BRCA1* carriers developed lung metastases as the first metastatic event (35% and 46%, respectively). On the contrary, in our series, no *BRCA2* carriers developed lung or liver metastases as first metastatic events. CNS involvement as the first metastatic event occurred in 32 (26.7%) patients. Among patients carrying a PV in *BRCA2*, three (60%) developed bone metastases, and two (40%) presented CNS involvement as the first event of distant dissemination. Among *BRCA1* carriers, 13% and 33.3%, respectively, showed the same evolution, while less frequent among noncarriers (18% and 25%, respectively) (*p* = 0.343). When a second metastatic event occurred, the involvement of multiple organs was the most prevalent form (Table 2).

**TABLE 2:** Visceral metastasis event by *BRCA* status (*N* = 388).

Variable	No. of patients (%)			
	*BRCA1* carriers	*BRCA2* carriers	*BRCA1/2* Noncarriers	*p*‐value
Visceral metastases				
Yes	15 (21.7)	5 (38.5)	100 (32.7)	0.173
No	54 (78.3)	8 (61.5)	206 (67.3)	
Time (months) between diagnosis and first visceral metastasis event—Mean (SD)	19.54 (11.6)	58.07 (77.0)	23.06 (27.0)	0.027*
First metastatic event				
Bone	2 (13)	3 (60)	18 (18)	0.343
CNS	5 (33.3)	2 (40)	25 (25)	
Liver	0 (0)	0 (0)	9 (9)	
Lung	7 (46.7)	0	35 (35)	
Other sites^a^	1 (6.7)	0	13 (13)	
Second metastatic event				
Bone	0	0	1 (1.6)	0.386
Liver	1 (7.7)	0	0	
Multiple (disseminated disease)	12 (92.3)	4 (100)	61 (98.4)	

Abbreviations: CNS, central nervous system; SD, standard deviation.

^a^ Mediastinum, ovary, skin, thyroid, etc.

* *p* < 0.05.

### 3.3 Bone or central nervous system metastasis according to *BRCA2* status

The number of the patients with TNBC who developed bone and CNS metastases during follow‐up period is shown on Table 3. There was difference in bone metastasis development among noncarriers and *BRCA1/BRCA2* carriers, but the same was not observed for CNS metastases. As expected, in both groups (patients with bone and CNS metastases), the stage of the primary tumor was statistically and significantly different according to the occurrence of the locoregional or systemic involvement of the disease.

**TABLE 3:** Number of patients who developed bone and central nervous system metastasis during follow‐up period.

Variables (*n*)^a^	Bone metastasis		*p*‐ value	CNS metastasis		*p*‐value
	Yes, *n* (%)	No, *n* (%)		Yes, *n* (%)	No, *n* (%)	
*BRCA* status (*n* = 388)						
Noncarrier	18 (79.0)	288 (78,9)	0.044*	25 (78.1)	281 (79.1)	0.616
*BRCA1* carrier	2 (8.7)	67 (18.4)		5 (15.6)	64 (18.0)	
*BRCA2* carrier	3 (13.0)	10 (2.7)		2 (6.3)	11 (3.1)	
Ethnicity (*n* = 286)						
Asian	1 (6.3)	9 (3.3)	0.473	0	10 (3.8)	0.664
Black	1 (6.3	10 (3.7)		1 (4.2)	10 (3.8)	
Brown	2 (12.5)	29 (10.7)		1 (4.2)	30 (11.5)	
White	12 (75.0)	222 (82.2)		22 (91.7)	212 (80.9)	
Tumor grade (*n* = 365)						
1	0	4 (1.2)	0.380	0		0.882
2	8 (38.1)	88 (25.6)		9 (29.0)		
3	13 (61.9)	252 (73.3)		22 (71)		
Histology (*n* = 382)						
Ductal	20 (87.0)	306 (85.2)	0.478	28 (90.3)	298 (84.9)	0.714
Lobular	1 (4.3)	7 (1.9)		1 (3.2)	7 (2.0)	
Mixed	2 (8.7)	26 (7.2)		1 (3.2)	27 (7.7)	
Other^b^	0	20 (5.6)		1 (3.2)	19 (5.4)	
Tumor classification (*n* = 373)						
T1	0	58 (16.5)	<0.0001***	0	58 (17.0)	0.002**
T2	6 (28.6)	196 (55.7)		14 (43.8)	188 (55.1)	
T3	5 (23.8)	45 (12.8)		7 (21.9)	43 (12.6)	
T4	6 (28.6)	47 (13.4)		10 (31.3)	43 (12.6)	
TX	4 (19)	6 (1.7)		1 (3.1)	9 (2.6)	
Lymph node classification (*n* = 373)						
N0	2 (9.5)	175 (49.7)	<0.0001***	6 (18.9)	171 (50.1)	0.001***
N1	8 (38.1)	101 (28.7)		12 (37.5)	97 (28.4)	
N2	2 (9.5)	43 (12.2)		6 (18.8)	39 (11.4)	
N3	3 (14.3)	21 (6.0)		7 (21.9)	17 (5.0)	
NX	5 (23.8)	11 (3.1)		1 (3.1)	15 (4.4)	
AJCC stage (*n* = 362)						
I	0	45 (13.2)	<0.0001***	0	45 (13.6)	0.001***
II	6 (27.3)	186 (54.7)		11 (36.7)	181 (54.5)	
III	9 (40.9)	92 (27.1)		17 (56.7)	84 (25.3)	
IV	7 (31.8)	17 (5.0)		2 (6.7)	22 (6.6)	
Neoadjuvant chemotherapy (*n* = 370)						
Yes	17 (73.9)	184 (53.0)	0.083	23 (71.9)	178 (52.7)	0.057
No	6 (26.1)	163 (47.0)		9 (28.1)	160 (47.3)	

^a^ Data available.

^b^ Metaplastic carcinoma, adenoid cystic carcinoma, spindle carcinoma, papillary carcinoma.

* *p* < 0.05

** *p* < 0.01

*** *p* < 0.001.

The *BRCA2* carriers tended to have a higher likelihood of developing bone metastases than noncarriers (OR, 4.06; 95% CI, 0.82–20.01; *p* = 0.085), and *BRCA1* carriers showed no trend (OR, 0.6; 95% CI, 0.12–2.87; *p* = 0.528). *BRCA2* carriers had an OR of 1.75 (95% CI, 0.33–9.14; *p* = 0.503) for CNS metastasis development when compared to noncarriers, while *BRCA1* carriers had an OR of 0.72 (95% CI, 0.23–2.23; *p* = 0.574), but this difference was not statistically significant. The other variables that differed in the univariate analysis did not represent independent factors for the development of bone or CNS metastases when analyzed in the multivariate model (Table 4).

**TABLE 4:** Multivariable logistic regression models for bone and central nervous system metastasis.

Variable	Comparison	Bone metastasis	*p*‐value	CNS metastasis	*p*‐value
		OR (95% CI lower‐upper)		OR (95% CI lower‐upper)	
BRCA status	*BRCA1* carrier vs noncarrier	0.60 (0.12–2.87)	0.528	0.72 (0.23–2.23)	0.574
	*BRCA2* carrier vs noncarrier	4.06 (0.82–20.01)	0.085	1.75 (0.33–9.14)	0.503
T (primary tumor size)	T3 vs T1/T2	1.96 (0.36–10.55)	0.434	1.46 (0.39–5.42)	0.572
	T4 vs T1/T2	2.90 (0.58–14.5)	0.193	1.71 (0.46–6.40)	0.420
N (regional lymph node)	(N1, N2, N3) vs (N0)	4.55 (0.87–23.89)	0.073	2.32 (0.75–7.13)	0.141
Initial stage	III vs I/II	1.01 (0.18–5.70)	0.982	1.72 (0.46–6.35)	0.413
	IV vs I/II	3.84 (0.62–23.57)	0.146	0.67 (0.10–4.57)	0.688
Neoadjuvant chemotherapy	No vs yes	1.43 (0.44–4.63)	0.544	1.39 (0.55–3.53)	0.482

Abbreviations: CNS, central nervous system; OR, odds ratio; vs, versus.

### 3.4 Risk of death according to *BRCA* mutation status and bone or central nervous system metastases

In multivariable analyses, both bone and CNS metastases were statistically significant being related to an increased risk of death (HR 3.29; 95% CI, 1.79–6.06; *p* < 0.0001 and HR, 6.49; 95% CI, 4.03–10.43; *p* < 0.0001, respectively) as well as advanced stage at the time of diagnosis. *BRCA2* carriers with bone or CNS metastases showed reduced risk of death (HR 0.44; 95% CI, 0.15–1.21; and HR, 0.80; 95% CI, 0.31–2.09, respectively), but these results were not significant (*p* = 0.114 and *p* = 0.659). *BRCA1* carriers with brain and bone metastases had a 48% reduced risk of death compared to noncarriers (HR, 0.52; 95% CI, 0.27–1.00; *p* = 0.053, Table 5). The median follow‐up was 75.69 months (95% CI, 64.01–87.37), and survival rate was 74%. More death events were recorded among noncarriers followed by *BRCA2* carriers, and *BRCA1* carriers (overall survival of 61.5%, 72.5%, and 82.6%, respectively) (*p* = 0.076; Figure 1B).

**TABLE 5:** Univariate and multivariable cox regression model for triple‐negative breast cancer death.

Variable	Category	Univariable Cox regression model		Multivariable Cox regression Model—bone metastasis				Multivariable Cox regression Model—CNS metastasis			
		HR (95% CI lower‐upper)	*p*‐value	HR (95% CI lower‐upper)			*p*‐value	HR (95% CI lower‐upper)			*p*‐value
Initial stage	IV vs I/II	4.47 (2.85–7.00)	<0.0001***	4.20 (2.66–6.63)			<0.0001***	3.61 (2.28–5.72)			<0.0001***
	III vs I/II	6.08 (3.34–11.07)	<0.0001***	4.07 (2.06–8.04)			<0.0001***	5.48 (2.95–10.17)			<0.0001***
BRCA status	*BRCA1* carrier vs noncarrier	0.50 (0.27–0.92)	0.028	0.52 (0.27–1.00)			0.053	0.52 (0.27–1.00)			0.053
	*BRCA2* carrier vs noncarrier	1.08 (0.43–2.68)	0.863	0.44 (0.15–1.21)			0.114	0.80 (0.31–2.09)			0.659
Bone metastasis	(M1) vs (M0)	5.26 (3.13–8.82)	<0.0001***	3.29 (1.79–6.06)			<0.0001***	—	—	—	—
CNS Metastasis	(M1) vs (M0)	8.74 (5.60–13.64)	<0.0001***	—	—	—	—	6.49 (4.03–10.43)			<0.0001***

Abbreviations: CNS, central nervous system; HR, hazard ratio; vs, versus.

*** *p* < 0.001.

## 4 DISCUSSION

The relation between germline PV in *BRCA1* and *BRCA2* genes and the development of visceral metastases has already been analyzed by other authors. However, those studies pooled together all BC subtypes.^1^, ^4^, ^17^ Among studies that investigated TNBC and PV in the *BRCA* gene, the biological behavior and natural history of the disease were analyzed mostly in relation to the *BRCA1* gene mutation (or in *BRCA1/2* carriers together), since patients with PV with the *BRCA2* gene and with TNBC are a very rare occurrence.^20^, ^21^, ^22^, ^23^, ^24^ BC among *BRCA1* carriers generally harbors more aggressive biological characteristics and are more commonly triple‐negative and preferably develop metastases to the lung and lymph nodes. On the contrary, PV of the *BRCA2* gene have a higher association with more indolent malignancies (luminal BC) and spread more frequently to the bones and liver.^7^, ^17^, ^25^, ^26^, ^27^

However, these studies evaluating the natural history of BC in *BRCA1* and *BRCA2* carriers usually investigate cases of all BC subtypes together and, due to the intrinsic association of *BRCA1* with TNBC and *BRCA2* with luminal BC subtypes, it is difficult to define the genuine interplay of the deficiency of each of these genes and the natural history of the disease, regardless of the intrinsic subtype. In this study, only TNBC was investigated, and all patients had germline status of *BRCA1* and *BRCA2* evaluated. As expected, it was possible to observe a lower mean age in the group of *BRCA1* carriers when compared to *BRCA2* carriers and noncarriers. The lower general age was understandable due to the inclusion of patients only with TNBC. This same characteristic may explain why there was no difference in the variables related to clinical and pathological characteristics between the three groups. Also, there was higher frequency of metastasis to the lung and CNS among *BRCA1* carriers as well as noncarriers. Patients with PV in the *BRCA2* gene, instead, had recurrences into the bone and the CNS.

Alternatively, if the patients that developed CNS metastases were considered, no difference was observed. In Song et al. study where an analysis was done using the multivariable logistic regression model, it was possible to verify an association between the *BRCA2* mutation and metastasis to the CNS; however, all the subtypes of BC were included in the analysis.^17^

Regarding the development of distant metastases in BC patients, TNBC has higher rates of systemic dissemination, and therefore an increased chance of developing bone and CNS metastases. This would also be expected for patients with HER2 overexpressing BC, but with current treatment regimens, employing the dual HER2 blockades, an improvement has been observed in disease‐free survival as well as an increase in survival rates following the diagnosis of CNS metastasis among HER2 BC patients.^28^

In this study, proportionally, more patients with germline PVs in the *BRCA2* gene developed visceral metastases, and the occurrence was only in the bones and the CNS. Furthermore, the metastatic occurrence was later, when compared to the other two groups (*p* < 0.05). This association was not demonstrated before considering an exclusive population of TNBC patients.^17^, ^19^, ^21^, ^24^, ^28^, ^29^

The main limitation of this study is the small number of *BRCA2* carriers, which is understandable given the rarity of *BRCA2*‐carriers who develop TNBC. Nonetheless in a population that included only TNBC cases, it was possible to find important associations, mainly regarding late recurrence presentation and bone and CNS preference of metastatic disease. In the TNBC subgroup from Song et al. paper, were evaluated 78 cases, 22 patients were *BRCA1* carriers, five patients were *BRCA2* carriers, and 51 patients were noncarriers.^17^

Bone metastasis is the most prevalent metastatic site in all BC subtypes, except in basal‐like BC, with 20% of patients with bone metastasis still being alive 5 years after diagnosis.^25^, ^30^, ^31^, ^32^ In TNBC nonbasal, the metastatic pattern is similar to that observed among Luminal and/or HER2 overexpressing subtypes.^32^ In the present study, TNBC was not classified between “basal‐like” and “non‐basal”, but upon analysis of the results, the overall clinical evolution of our cohort seems more associated with the first subtype. Pogoda et al. evaluated pattern, time, and risk factors influencing recurrence in 228 TNBC patients, and described that bone metastasis occurred in 11% of the patients, during the follow‐up period. In addition, the risk of bone metastasis was higher after 5 years of observation, which influenced the overall survival of patients. In the same study, the most common sites of recurrence were brain and lungs.^33^ Klimov et al. described an immunohistochemistry‐based signature to predict the metastatic site of TNBC, that was able to stratify TNBC patients into high‐risk groups of developing metastases to the bone, liver, lung, and brain. In the multivariable analysis, this model was more accurate in predicting bone metastases in patients with TNBC than the size of the primary neoplasia, systemic therapy employed, and age at diagnosis (HR 4.93; 95% CI, 2.28–10.69; *p* < 0.0001).^34^ In the study by Song et al., a higher prevalence of bone involvement was found, as the first metastatic event, in *BRCA2* carriers (56%), when compared *to BRCA1* carriers (27%) and noncarriers (38%). This may have occurred due to the presence of luminal BC cases in the sample evaluated.^17^

Only lung cancer has more metastases to the CNS when compared to BC. In autopsy studies from the 70s and the 80s, CNS metastases were described in up to 30% of BC patients.^35^ If all immunohistochemical subtypes of BC are considered, the CNS metastasis rates vary from 5% to 15%, and, about 80% of the cases occur after the diagnosis of metastasis in anatomical sites.^36^ CNS metastasis is considered a later event during BC progression. It occurs 2 or 3 years after initial diagnosis, and in general is preceded by lung, liver or bone metastases.^19^, ^37^

Risk factors for the development of CNS metastasis in BC patients are advanced age, and, HER2 and TNBC subtypes.^19^, ^34^, ^38^, ^39^, ^40^, ^41^ In 2010, Graesslin et al. published a nomogram to predict the chance of developing brain metastasis in female BC patients, but the presence of germline PV in *BRCA1/2* was not included in the variables evaluated. However, the variables that contributed most to the chance of developing CNS metastasis were young age, histological grades 2 and 3, short interval between BC diagnosis and first systemic recurrence, greater number of metastases, HER2 receptor overexpression and TNBC. There was no association with disease stage at diagnosis or with ethnicity.^36^ In this study, variables related to a worse prognosis such as disease stage at diagnosis and characteristics of the primary neoplasm did not independently increase the chance of developing bone or CNS metastasis. The same occurred when self‐reported ethnicity was analyzed. However, the self‐declared ethnicity does not often correspond to the ancestry of the Brazilian mtDNA lineages, considering that the Brazilian population is one of the most admixed in the world.^42^

When the variables related to disease‐free survival were analyzed, *BRCA1* carriers presented systemic recurrence earlier when compared to the other two groups and, despite this, there was tendency to higher overall survival. In studies where only *BRCA1* carriers with TNBC were evaluated, this association also presented a better prognosis.^28^ Conversely, in another study, where worse survival was observed in *BRCA1* and *BRCA2* carriers, regardless of the subtype of BC, the authors correlated the results with worse prognostic variables, but the number of *BRCA1/2* carriers was small (*BRCA1* = 16 and *BRCA2* = 4).^43^ Similarly, in a systematic review and meta‐analysis from the KOHBRA Research Group, *BRCA1* carriers presented worse prognoses when compared to *BRCA2* carrier and noncarrier groups.^44^ Other meta‐analyses, however, did not observe better survival among *BRCA1* carriers.^45^, ^46^

## 5 CONCLUSION

In TNBC patients associated with germline PV in the *BRCA2* gene, there was a higher number of cases with bone and CNS metastases, although it does not represent an independent risk factor for the spread of the disease to the brain or meninges and bone. Moreover, in this group of patients, the metastatic events occurred later. Additionally, it was possible to verify that patients with TNBC and PV in the *BRCA1* gene showed fewer metastatic events at an earlier age and had a better survival rate when compared to the *BRCA2*‐carriers and noncarriers. Since TNBC patients with PV in the *BRCA2* gene are a rare event, efforts to increase the number of cases from different centers are crucial to better characterize the pattern of metastasis and survival rate in this population.

## AUTHOR CONTRIBUTIONS

**Marcelo Moreno:** Formal analysis (equal); writing – original draft (equal); writing – review and editing (equal). **Júlia Salles Oliveira:** Investigation (equal); writing – original draft (equal); writing – review and editing (equal). **Rafael Canfield Brianese:** Investigation (equal); validation (equal); writing – review and editing (equal). **Douglas Guedes de Castro:** Conceptualization (equal); writing – review and editing (equal). **Solange Moraes Sanches:** Data curation (equal). **Giovana Tardin Torrezan:** Investigation (equal); validation (equal); writing – review and editing (equal). **Karina Miranda Santiago:** Investigation (equal); writing – review and editing (equal). **Marina De Brot:** Data curation (equal). **Vladmir Claudio Cordeiro de Lima:** Conceptualization (equal); writing – review and editing (equal). **Fabiana Baroni Alves Makdissi:** Data curation (equal). **José Claudio Casali‐da‐Rocha:** Data curation (equal). **Vinicius Fernando Calsavara:** Formal analysis (equal). **Dirce Maria Carraro:** Conceptualization (lead); funding acquisition (lead); resources (lead); writing – original draft (equal).

## FUNDING INFORMATION

This work was funded by The São Paulo Research Foundation (FAPESP 2014/50943‐1 and 2013/23277‐8); National Council for Scientific and Technological Development (465,682/2014‐6); and Coordination for the Improvement of Higher Education Personnel (CAPES–88887.136405/2017‐00).

## CONFLICT OF INTEREST STATEMENT

The authors declare no potential conflict of interest.

## ETHIC APPROVAL

This study was approved by the ACCCC Institutional Review Board (CAAE 12601013.3.0000.5432 and 16580619.6.0000.5432).

## PATIENT CONSENT STATEMENT

All study participants provided written informed consent, either specific for the genetic testing or for Biobank collection.

## Data Availability

All data are available in the manuscript or supplementary materials.
